# Ubiquitin specific protease 7 is a potential therapeutic target for gastric cancer

**DOI:** 10.3389/fonc.2025.1530924

**Published:** 2025-02-21

**Authors:** Zhi-Ru Wang, Wen-Ting Kang, Rui-Li Yu

**Affiliations:** ^1^ Department of Pathology, Henan Provincial People’s Hospital, People’s Hospital of Zhengzhou University, People’s Hospital of Henan University, Zhengzhou, Henan, China; ^2^ Clinical Operations Department, Jiangsu Hengrui Pharmaceuticals Co., Ltd, Nanjing, Jiangsu, China

**Keywords:** USP7, gastric cancer, proliferation, prognosis, p53

## Abstract

**Introduction:**

As a deubiquitinase, ubiquitin-specific protease 7 (USP7) plays a vital role in diverse cancers, nevertheless, its role in gastric cancer (GC), which is the fifth leading cause of death in diverse cancers worldwide, has rarely been reported.

**Methods:**

To gain a comprehensive understanding about USP7 in the progression of GC, 287 paired GC tissues were collected and analyzed. ShRNA and small molecular inhibitor were used to investigate the impact of USP7 on cell proliferation. Additionally, xenograft proliferation was used to explore the effect of USP7 on tumor growth in animals.

**Results:**

We found that USP7 overexpression in GC tissues was correlated with several parameters of clinicopathology and poor disease-free survival rate. Meanwhile, the abrogation of USP7 suppressed GC cell proliferation by stabilizing p53 and promoting cell cycle arrest both in vitro and in vivo.

**Discussion:**

These results indicate that USP7 may serve as a biomarker for predicting the outcomes of patients with GC. Therefore, USP7 could be a promising therapeutic target for GC treatment.

## 1 Introduction

GC is highly lethal and ranks as the fifth most frequently diagnosed cancer globally and the fifth leading cause of cancer death. The prognosis for patients with GC is poor and the 5-year survival rate is <10% (1). Nevertheless, due to the limited understanding of GC pathogenesis and the shortage of targeting therapy, available remedies like chemotherapy and surgical operation do not satisfactorily improve the survival rate of GC patients (2), although some studies have indicated that Her2, E-cadherin, fibroblast growth factor receptor as well as microRNAs, and long noncoding RNAs, can be GC markers (3). Therefore, it is necessary to comprehensively understand the pathogenesis and biological features of GC.

The ubiquitin-proteasome system has a significant impact on the regulation of cellular processes in eukaryotic cell, including advancement of cell cycle, response to stress response, and cellular signaling (4). In this system, proteins tagged with ubiquitin can be degraded. Meanwhile, a series of deubiquitinases can erase the ubiquitin and rescue the modified protein from degradation.

USP7, which is also known as herpesvirus-associated ubiquitin-specific protease (HAUSP), was initially recognized as a herpesvirus-associated cellular factor (5)and subsequently proved to deubiquitinate and stabilize p53 (6). To date, several studies have shown that USP7 targets several crucial regulatory proteins to enhance their stability or subcellular location, such as phosphatase and tensin homolog (PTEN) (7), mediator of DNA damage checkpoint protein 1 (MDC1) (8), Programmed cell death ligand 1(PD-L1) (9), Epstein-Barr virus nuclear antigen 1(EBNA1) (10), and murine double minute-2 (MDM2) (11), which act as tumor suppressors, DNA repair proteins, immune responders, viral proteins, and epigenetic modulators. Hence, the relationship between USP7 and cancer progression is remarkable. For example, in lung cancer, overexpression of USP7 is directly associated with non-small-cell lung cancer (NSCLC) cell glycolysis and survival (12). Furthermore, another study claimed that USP7 deubiquitinates KRAS, stabilizing it and promoting the proliferation of NSCLC cells (13). USP7 can also deubiquitinates estrogen receptor (ERβ) to promote osimertinib resistance in NSCLC cells (14). The acquired knowledge based on the above findings from different research groups implies that the possible role of USP7 in GC needs to be clarified.

In our study, a series of data contributed to the carcinogenic and non-negligible role of USP7 in GC. This study was performed with a view to analyze the expression level of USP7 in clinical and cell lines to study its role in GC tumorigenesis and assess the probability of USP7 as a target for the therapy of GC.

## 2 Materials and methods

### 2.1 Cell culture

The human gastric cancer cell lines BGC-823, MGC-803, HGC-27, and MKN45 were obtained from the Cell Bank of the Chinese Academy of Sciences (Shanghai, China). NCI-N87, SGC-7901 and GES-1 were obtained from the Cell Bank of the Chinese Academy of Sciences, Beijing, China. The cells were maintained in the corresponding medium supplemented with 10% fetal bovine serum (FBS) at 37°C with 5% CO_2_.

### 2.2 Protein extraction and immunoblotting

To prepare samples for immunoblotting, protein was extracted from cells with Radioimmunoprecipitation lysis buffer containing protease inhibitor to protect protein degradation. Cells were washed with phosphate buffer saline (PBS), mixed with lysis buffer and then centrifugation was applied at 12,000×g for 10 minutes at 4°C. The supernatant was collected and the protein concentration was measured using a Bicinchoninic Acid (BCA) Protein Assay Kit. Following, denatured at 100°C for 10 min, samples were subjected to SDS-PAGE, and then protein was transferred to 0.22 μm nitrocellulose membranes. After blocking with PBS containing 5% skim milk for 2h at room temperature, the membranes were probed with specific primary antibodies diluted in PBST at 4°C overnight, followed by treatment with a horseradish-peroxidase-conjugated secondary antibody at room temperature for 2h. Subsequently, the membranes were washed with PBST, and exposed to an enhanced chemiluminescence kit (Thermo Fisher Scientific, Rockford, IL). The antibodies used for this study were against USP7 (Abcam No. 10893), MDM2 (Cell Signaling Technology No. 86934S), p53 (Cell Signaling Technology No. 9282T), and p21 (Cell Signaling Technology No. 2946T), GAPDH (Goodhere No. AB-M-M 001), Nanog (Abcam No,21624), E-cadherin (Cell Signaling Technology No. 3195S), ZO1 (Cell Signaling Technology No. 8193S), Vimentin (Cell Signaling Technology No. 5741S).

### 2.3 Immunohistochemical analysis

Gastric carcinoma tissues were acquired from the Henan Provincial People’s Hospital. Prior to tissue collection, the clinical protocol was approved by the Ethics Review Board of the Henan Provincial People’s Hospital. Written informed consent was obtained from all patients. Every specimen was handled anonymously, according to ethical and legal standards. Deparaffinization of the slides containing histological samples were performed by xylene and different concentrations of ethanol and washed with PBS three times. Then the slides were incubated with 3% hydrogen peroxidase (H_2_O_2_) for 20 min at room temperature to block endogenous peroxidase, then rinsed in PBS, and blocked with 5% BSA. Anti-USP7 antibody was applied to the sections at 4°C overnight. After washing three times with PBS, the slides were incubated with the secondary antibody for 2h at 37°C, subsequently, the slides were stained with DAB and hematoxylin. Finally, the slides were dehydrated and mounted. The slides were scanned and analyzed using Aperio AT2 (Leica).

### 2.4 shRNA treatment

Cells were seeded in 96-well plate at approximately 30% confluency for 24h before shRNA transfection. After transfection with Lipofectamine 3000, the medium was replaced with fresh medium. Three days later, cells were harvested and used for the indicated experiments. The following USP7 shRNA sequence was used: 5’- AAGGACCCGCAAAA-3’ (15).

### 2.5 Colony formation assay

1×10^3^ transfected cells of BGC-823 and MKN45 cells were seeded in each well of 6-well plate in triplicate. 1×10^3^ MKN45 cells were incubated in 6-well plate with or without Almac4 (0, 2.5, 5, 10μM) for 24h. After that, the medium was replaced with fresh medium, and the cells were cultured for an additional 10 days. Formed colonies (>50 cells/colony) were fixed and stained with methanol containing1% crystal violet (Beyotime Biotechnology, Haimen, China) and colony number was counted using Image J software (National Institutes of Health). Three independent experiments were performed to collect data, and the results were presented as the mean ± SD.

### 2.6 Cell proliferation assay

The equal number of the BGC-823 and MKN45 cells (1×103 cells per well) were seeded in a 96-well plate after transfection in triplicate. One thousand MKN45 cells were seeded in 96-well plate with or without Almac4 (0, 6.25, 12.5, 25, 50μM). At 1, 2, 3, 4, 5, 6, and 7 days, each well was added with 20μl of MTT [3-(4,5-dimethylthiazol-2-yl)-2,5-diphenyltetrazolium bromide] (obtained from Solarbio, Beijing, China) solution (5mg/ml) and then incubated at 37°C for 4h. Then 200 μL of dimethyl sulfoxide (DMSO) was added to dissolve formed formazan crystals, and the absorbance was taken at 570 nm detected on a microplate reader (Perkin Elmer).

### 2.7 Cell cycle analysis by flow cytometry

The cells were harvested, centrifuged, and fixed with ice-cold 70% ethanol at 4°C overnight. Then cells were rinsed with PBS before staining with Propidium Iodide (PI) and suspended in staining buffer (50 μg/ml PI, 1% Triton X-100 and 0.5 mg/ml RNase in PBS) at 37°C for 30 min in the dark. Finally, the cells were analyzed using a BD Calibur flow cytometer (BD, USA). The results were analyzed using FlowJo V7.6 software (TreeStar, USA). DNA distribution at G0/G1, G2/M, or S phase was analyzed using FlowJo software and presented as histogram.

### 2.8 Animals and tumor xenograft model

All animal experiments were conducted in accordance with the guidelines of the Institutional Animal Care and Use Committee of the Zhengzhou University. Female BALB/c nude mice of age range from four- to five-week-old with weighing 18-21g, were purchased from Hunan Slack Scene of the Laboratory Animal Company, LTD (Hunan, China). Optimum food and water were supplied to each animal and the nursing was done under sterilized conditions. The right scapular region of mice was selected and the cancer cells (5×10^6^) were injected. The weight of mice and the tumors were measured every 3 days. On the 18th day, the tumors were obtained from mice and weight was measured. The size of tumor was measured using vernier caliper.

### 2.9 Statistical analyses

Data was presented as the mean ± Standard Deviation. The significance of the difference between different groups was calculated using analysis of variance (ANOVA) and Student’s *t*-test. Student’s *t*-test was used to compare the difference between the two groups using SPSS 17.0. Results were regarded statistically significant at 0.01<p<0.05. p<0.01 was regarded highly significant.

## 3 Results

### 3.1 USP7 was overexpressed in GC tissues and correlated with GC clinicopathological and survival characteristics of GC patients

**Figure 1:**
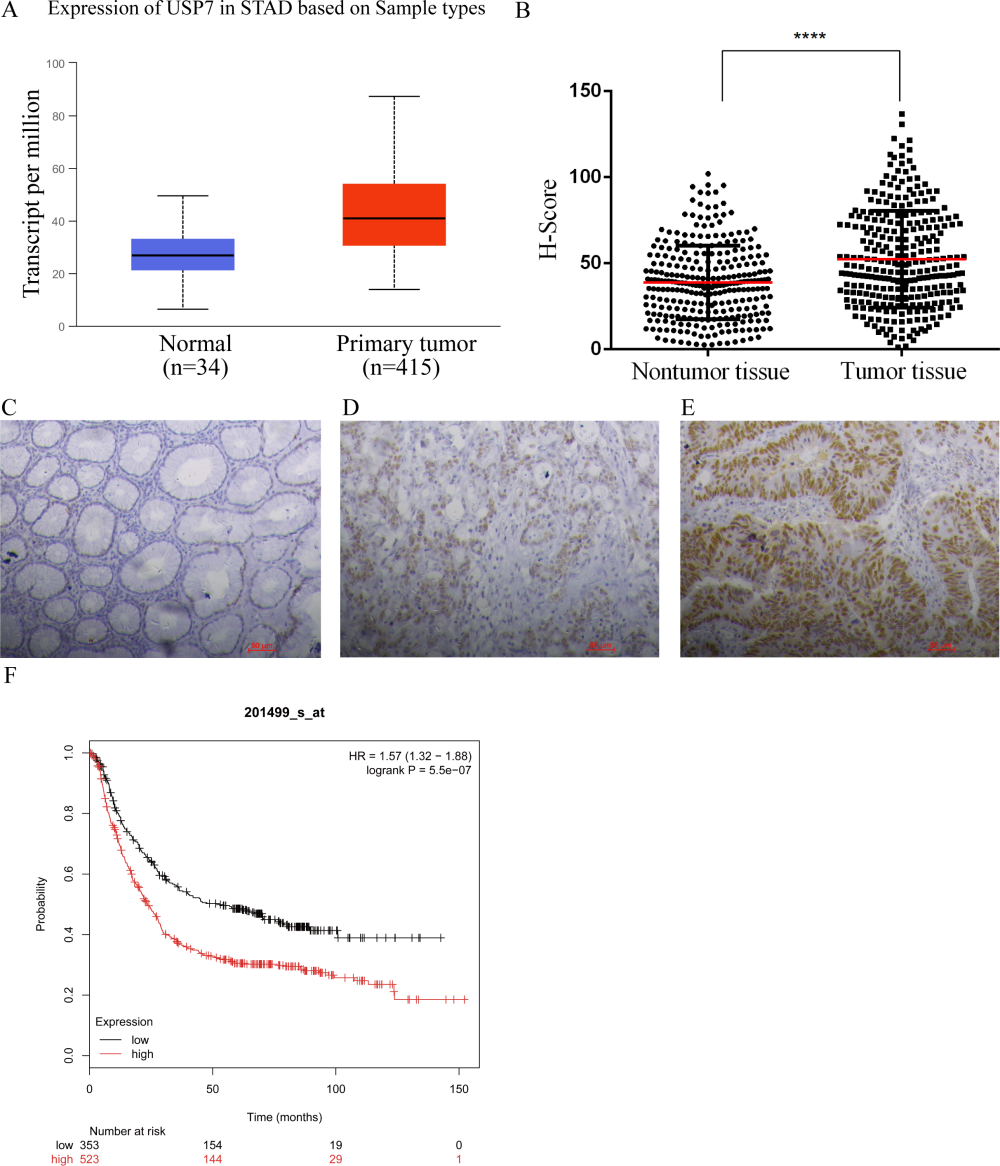
USP7 was overexpressed in GC tissues. **(A)** Expression of USP7 analyzed by UALCAN in gastric adenocarcinoma according to TCGA data (http://ualcan.path.uab.edu), the statistical significance was 2.8E-11; **(B)** Expression of USP7 in 287 paired human GC tissues and paired adjacent normal tissues, p-values were calculated using two-tailed t-test and “****” was considered as p<0.001; **(C-E)** Representative IHC results of USP7 in normal gastric tissues **(C)**, weak **(D)** and high **(E)** protein levels in GC tissues; **(F)** Overall survival analysis conducted by Kaplan-Meir method according to USP7 expression (http://kmplot.com).

First, UALCAN was used to evaluate the expression level of USP7 in GC tissues (16). **Figure 1A** showed that USP7 was overexpressed in GC tissues compared to that in normal gastric tissues. To further confirm this finding, the expression of USP7 in 287 human GC tissues with adjacent normal tissues was examined in our in-house tissue library, and the results showed that the expression of USP7 in tumor tissues was higher than that in adjacent normal tissues (**Figures 1B–E**), which is consistent with the finding from UALCAN. After confirmation the upregulation of USP7 in GC tissues, the clinical relevancy of USP7 with GC progression was investigated. As shown in **Table 1**, USP7 expression was correlated with the stage of differentiation (p = 0.011), but not with age, gender and metastasis in GC patients. In addition, Kaplan-Meier analysis revealed that patients with high USP7 expression had significantly worse overall survival than those with low USP7 expression (**Figure 1F**). Overall, these data indicated that USP7 was overexpressed in GC and that USP7 overexpression may contribute to the poor survival outcome of GC patients.

**Table 1:** Clinical characteristic of GC patients with different USP7 expression.

Variables	NO. of patients	USP7 expression		p value
		low	high	
Age				
<51	48	24	24	0.552
≥51	239	120	119
Gender				
Male	217	110	107	0.785
Female	70	34	36
Lymph node Metastasis				
Yes	201	103	98	0.335
No	86	41	45
Differentiation				
Well/moderate	132	56	76	0.011
Poor	155	88	67

### 3.2 Abrogation of USP7 suppressed GC cell proliferation and cell cycle progression

**Figure 2:**
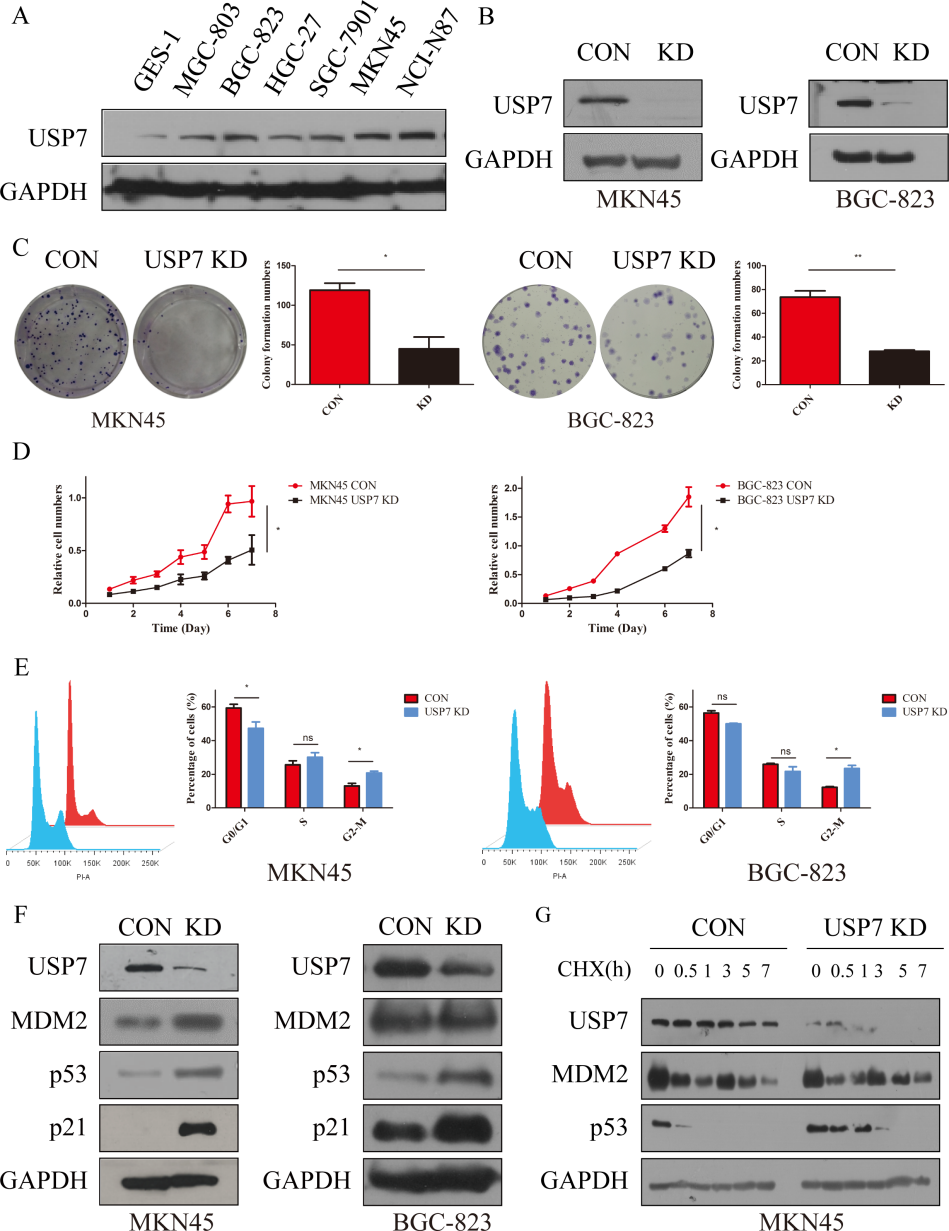
Abrogation of USP7 suppressed GC cell proliferation and cell cycle progression. **(A)** USP7 expression in six GC cell lines and one normal gastric epithelial cell line; **(B)** Verified USP7-shRNA mediated interference of USP7 expression in MKN45 and BGC-823 cells; **(C)** Colony formation capacity of CON and USP7 KD cells; **(D)** Growth curves of MKN45 and BGC-823 USP7 KD cells compared with control cells; **(E)** Cell cycle analyses of MKN45 and BGC-823 CON and USP7 KD cells; **(F)** Expression of p53 and p21 in CON and USP7 KD cells; **(G)** Turnover of p53 on USP7 knockdown; Data reported as the mean of at least three independent experiments with s.d. *p ≤ 0.05; **p ≤ 0.01, two tailed paired t test. “ns” stands for “no significant”.

As USP7 was confirmed to be overexpressed in GC, role of USP7 in the development and progression of GC remains unexplored. Hence, the expression of USP7 in seven gastric cancer cell lines was investigated. Results in **Figure 2A** showed that six human GC cell lines express high amount of USP7 comparing with the normal gastric epithelium cell line GES-1. Among these cell lines, MKN45 and BGC-823 were chosen to explore the function of endogenous USP7 in cell proliferation due to their high expression of USP7. Once USP7 was knocked down (**Figure 2B**), the colony formation efficiency of MKN45 and BGC-823 cells was inhibited compared to that in control cells (**Figure 2C**). What is more, proliferation of MKN45 and BGC-823 cells was also inhibited when USP7 was abrogated (**Figure 2D**). In the wound healing assay, knockdown USP7 decreased healing of wounds in MKN45 cells compared with the control group (**Supplementary Figure S2A**). Another study also found that the USP7 inhibitor slowed the closure of the would relative to the control group in MGC803 cells (17). These data indicated that knockdown of USP7 reduced colony formation ability and suppressed the proliferative ability on MKN45 and BGC-823 cells.

As reported, the TRAF domain and C-terminal (amino acids 801-1050) of USP7 interact with MDM2, which contributes to its stability through erasing its tagged ubiquitin, and protecting it from proteasome degradation (11). Meanwhile, MDM2 is the key E3 ligase of p53 and can write a poly-ubiquitin tag on p53 for subsequent 26S proteasome-dependent degradation (18). In contrast, the CDK inhibitor p21 was the first identified transcriptional target for p53. Once p53 is activated, p21 is transcriptionally upregulated via direct binding of p53 to the p21^CDKN1A^ promoter. Then p21 inhibits cyclin-dependent kinases (CDKs) activity, which phosphorylate the pRB-related proteins p107 and p130, and induces cell cycle arrest to inhibit cell viability (19). Thereafter, impact of USP7 on cell cycle was tested and results showed that abrogation of USP7 in MKN45 and BGC-823 cells promotes cell cycle arrest at G2/M phase (**Figure 2E**), while knockdown of USP7 has no effect on MKN45 cells apoptosis (**Supplementary Figure S1A**). To further characterize the molecular mechanisms which govern G2/M arrest as a result of USP7 abrogation, the expressions of p53 and p21 in MKN45 and BGC-823 cells were investigated, and the results showed that both proteins accumulated upon knockdown of USP7 (**Figure 2F**). After seeing that USP7 knockdown inhibits MKN45 cell migration, we investigated the status of mesenchymal marker proteins. The results showed that knockdown USP7 reduced the level of the mesenchymal marker Vimentin in MKN45 cells (**Supplementary Figure S2B**). A similar downregulation of Vimentin was observed when USP7 was knockdown in HCT116 cells (20). To characterize the accumulation mechanism of p53 upon USP7 abrogation, half-life of p53 was investigated and results indicated that abrogation of USP7 can stabilize p53 (**Figure 2G**, **Supplementary Figures S1B, C**). All these data collectively indicated that USP7 is involved in cell cycle progression by promoting the stability of p53.

### 3.3 Inhibition of USP7 suppressed GC cell proliferation and cell cycle progression

**Figure 3:**
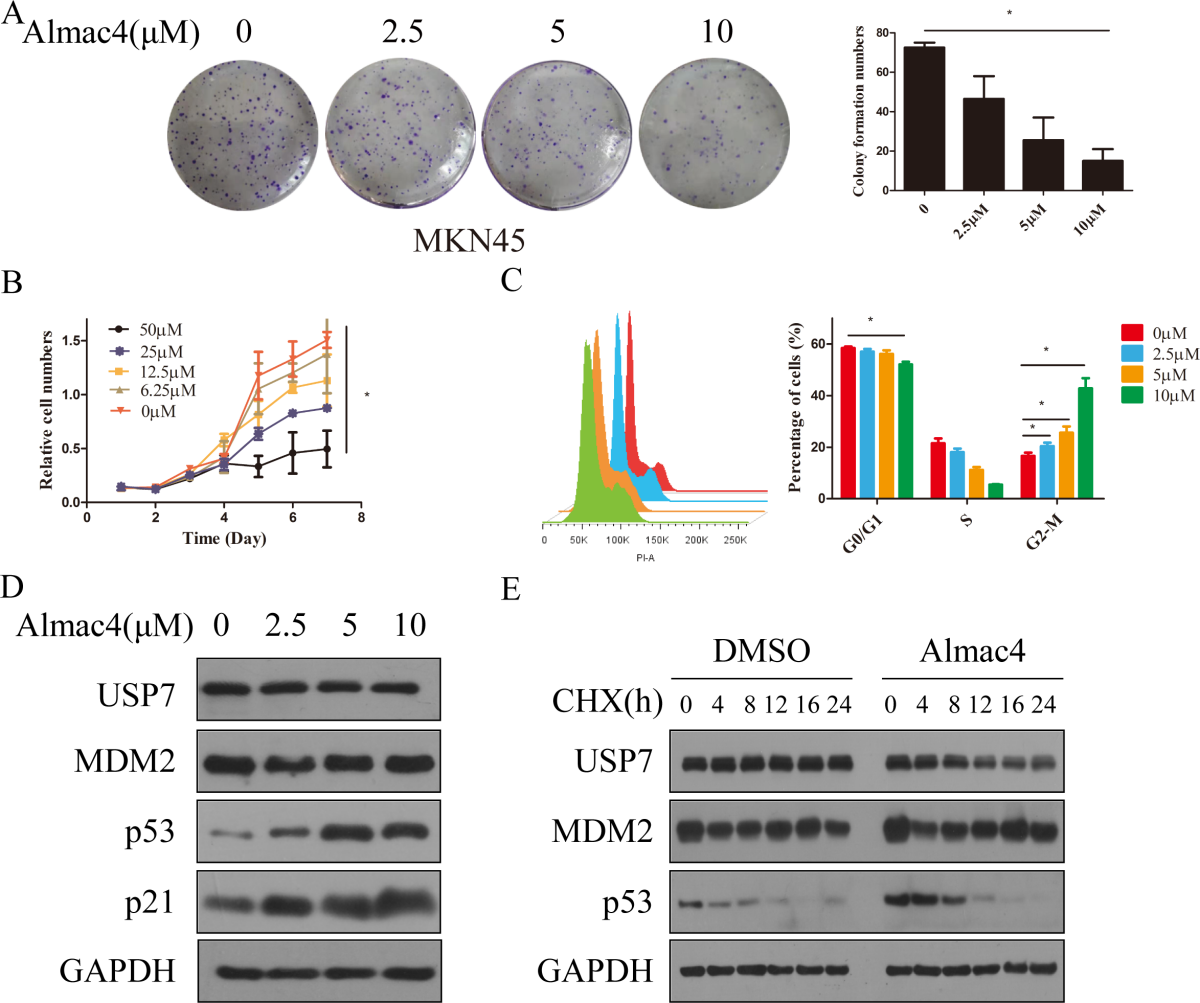
Inhibition of USP7 suppressed GC cell proliferation and cell cycle progression. **(A)** Colony formation of MKN45 cells treatment with different concentration of Almac4; **(B)** Growth curves of MKN45 cells treated with different concentration of Almac4; **(C)** Cell cycle analyses of MKN45 cells treated with different concentration of Almac4; **(D)** Expression of p53 and p21 in MKN45 treated with Almac4 **(E)**Turnover of p53 on USP7 inhibition. Data reported as the mean of at least three independent experiments with S.D. *p ≤ 0.05, two tailed paired t test.

Based on the results above, although USP7 was confirmed as a promoter in GC progression, whether USP7 is a drug target in GC still remains to be answered. Hence, USP7 inhibitor Almac4 was applied to verify the USP7 function on cell proliferation and colony formation, and results showed that Almac4 decreased the colony formation and proliferation activity of MKN45 cells in a dose-dependent manner (**Figures 3A, B**). In addition, cell cycle analysis uncovered that cell numbers accumulate in the G2/M phase in the presence of Almac4 in a dose dependent manner either (**Figure 3C**). Further pharmacological inactivation using Almac4 also induced the expression of p53 and p21 in MKN45 cells in a dose-dependent manner (**Figure 3D**). To further explore the accumulation mechanism of Almac4 on p53, half-life of p53 was tested and results suggested that Almac4 reduced endogenous p53 turnover in the presence of cycloheximide (**Figure 3E** & **Supplementary Figure S1D**). These data revealed that USP7 inhibitor suppresses GC cell proliferation and cell cycle progression.

### 3.4 USP7 knockdown suppressed GC tumor growth *in vivo*

**Figure 4:**
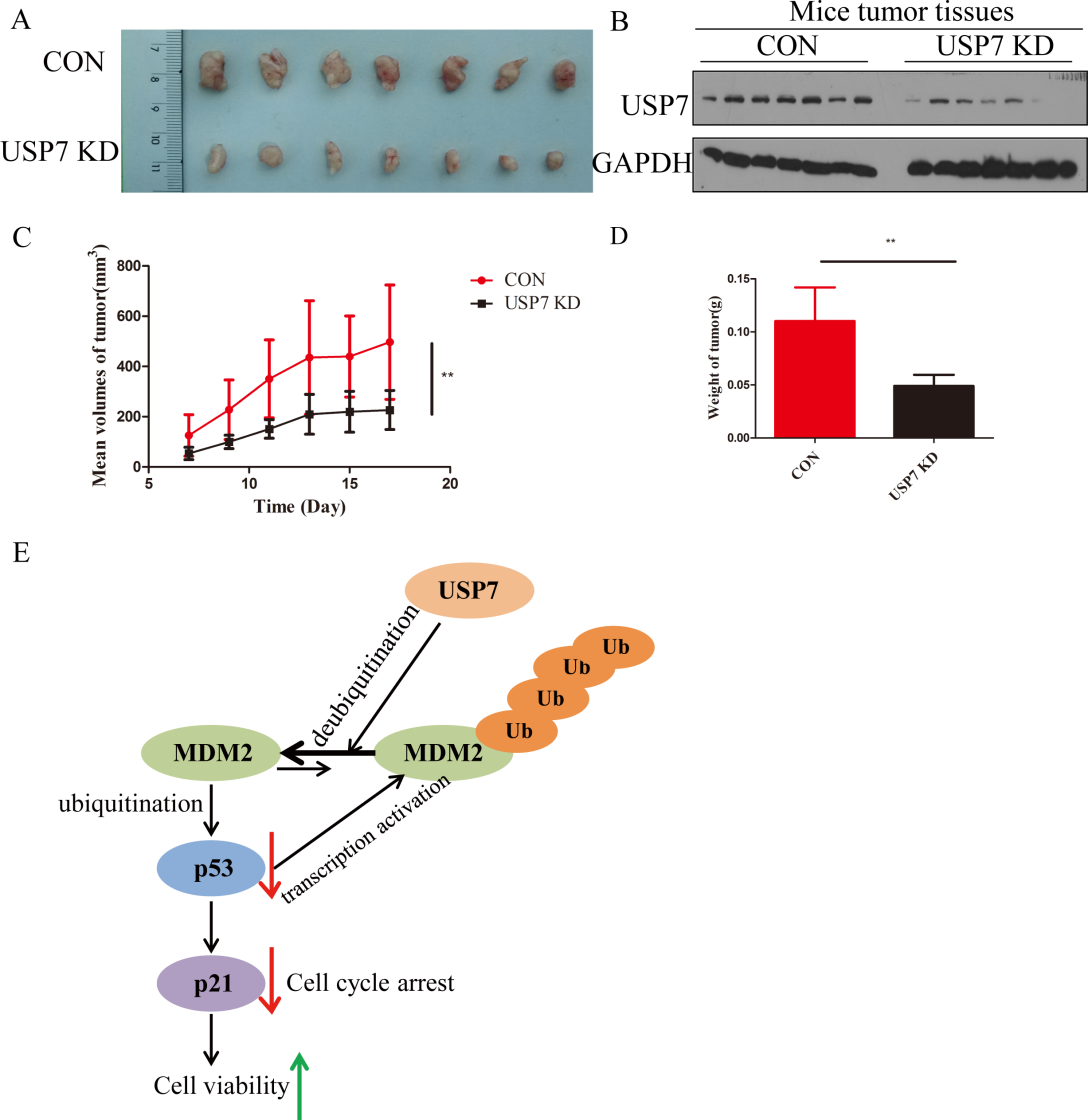
USP7 promoted GC tumor growth *in vivo*. **(A)** Representative tumors formed in nude mice of CON and USP7 KD MKN45 cells; **(B)** Expression of USP7 in xenograft model bearing CON and USP7 KD MKN45 cells; **(C)** The tumor volume (average ± SD) in xenograft model bearing CON and USP7 KD MKN45 cells; **(D)** The tumor weight in xenograft model bearing CON and USP7 KD MKN45 cells. **p ≤ 0.01, two tailed unpaired *t* test. **(E)** Illustration of USP7 signaling through the MDM2-p53-p21 axis.

Above results let us make a hypothesis that knockdown of USP7 can suppress GC tumor growth *in vivo*, the ability of USP7-knockdown cells to affect tumor growth was detected in xenograft model bearing Con and USP7 KD MKN45 cells. The results in **Figure 4A** showed that knockdown of USP7 in MKN45 cells significantly reduced tumor size compared to the control group. Besides, the expression levels of USP7 in tumors were tested and lower USP7 protein levels are detected in the USP7 knockdown group compared to control group in general (**Figure 4B**). At the same time, abrogation of USP7 decreased the tumor volume and weight in the USP7 KD group compared to the control group, and the difference between these two groups was significant (**Figures 4C, D**). Overall, these results suggested that the abrogation of USP7 can restrict the proliferation of tumor cells *in vivo*.

## 4 Discussion

GC overexpresses ubiquitin-specific proteases (USPs), which are significant members of the deubiquitinating enzyme family. USPs stimulate GC proliferation, invasion, metastasis, and epithelial-mesenchymal transition by controlling several signaling pathways and encouraging the production of proteins linked to deubiquitination and stability (21). Accumulated evidence uncovers the importance of USP7 in controlling cell death and proliferation, and regulating pivotal biological signaling pathways in human during tumorigenesis (22–24). Notably, this is the first study to prove that USP7 is a potential therapeutic target for GC.

Firstly, with data from TCGA and our in-house tissue library, USP7 was found to be significantly overexpressed in GC tissues than that in adjacent normal tissues. On the basis of the above results, this study uncovered that the expression of USP7 is related with the stage of differentiation in GC. In addition, Kaplan-Meier analysis indicated that high USP7 expression was associated with poor overall survival. However, only 287 pairs of gastric cancer tissues and adjacent normal tissues were used in this study, and only the correlation between USP7 expression and age, gender, lymph node metastasis, and degree of differentiation was analyzed. The small sample size did not allow us to analyze the relationship between USP7 expression and molecular staging of GC. We will further analyze the correlation between USP7 expression in various subtypes of GC and the response to neoadjuvant chemotherapy using TCGA database and local samples. All in all, these data revealed the prognostic value of USP7 as a novel biomarker of GC progression.

As USP7 is participated in a dynamic regulation with MDM2 and p53, overexpression of USP7 was shown to stabilize p53 (15), however, genetic blockage of USP7 produced a similar effect because of the destabilization of MDM2 (6). Secondly, our study established that USP7 stabilizes MDM2 to reduce expression of p53 in GC, allowing for cellular proliferation (**Figure 4E**). This current study is limited by the fact that it only used one USP7 shRNA. It may pose the risk of insufficient knockdown efficiency or off-target effects. Although we observed an increase in p53 expression after USP7 knockdown, these results should be further validated using multiple shRNAs or in combination with the CRISPR-Cas9 technology. It is unfortunate that the study did not include the overexpression of USP7. To verify that the USP7 gene directly regulates these alterations, functional experiments including overexpression of USP7 should be conducted in future research. Thirdly, knockdown of USP7 suppressed GC cell growth *in vivo*. These results confirmed that knockdown of USP7 suppressed tumor growth partly mediated by p53.

As the co-crystal structure of USP7 and small molecule inhibitor was not available, there has been no potent and selective USP7 inhibitor for a long time. Nevertheless, in 2017, two groups filled this gap and reported the complex structures of USP7 with small-molecule inhibitors at the same time (15). This study’s drawback is that insufficient amounts of the Almac4 prevented the function of USP7 inhibitor in nude mice from being confirmed. However, Xiaoqing Guan and colleagues discovered DHPO, a new USP7 inhibitor. The effectiveness of DHPO in preventing tumor growth and spreading without causing appreciable damage was confirmed by *in vivo* investigations employing an orthotopic gastric tumor mice model (17). In addition, Christopher Chen et al. discovered that GNE6776 decreased the formation of EBV+ tumors in a mouse xenograft model and specifically suppressed the proliferation of EBV+ gastric and lymphocytes in cell culture (10). These studies highlight the potential of USP7 for GC treatment.

## 5 Conclusions

In a nutshell, pharmacological and genetic abrogation of USP7 can inhibit the proliferation of MKN45 cells both *in vitro* and *in vivo*, which indicates that USP7 can be considered as a promising therapeutic target for the treatment of gastric cancer.

## Data Availability

The original contributions presented in the study are included in the article/**Supplementary Material**. Further inquiries can be directed to the corresponding author.
