# Supplementary material for: Ubiquitin specific protease 7 is a potential therapeutic target for gastric cancer
Source: Front Oncol. 2025 Feb 21;15:1530924. doi: 10.3389/fonc.2025.1530924 (PMC11885118; doi:10.3389/fonc.2025.1530924)
Supplement: Supplementary file 1 [file DataSheet1.docx]

**Supplementary Information for**

**Original Article**

Ubiquitin specific protease 7 is a potential therapeutic target for gastric cancer

Zhi-Ru Wang^1^, Wen-Ting Kang ^2^，Rui-Li Yu^1^

1 Department of Pathology, Henan Provincial People's Hospital, People's Hospital of Zhengzhou University, People's Hospital of Henan University, Zhengzhou, Henan, 450001, China

2 Jiangsu Hengrui Pharmaceuticals Co., Ltd, Nanjing, Jiangsu, 222002, China


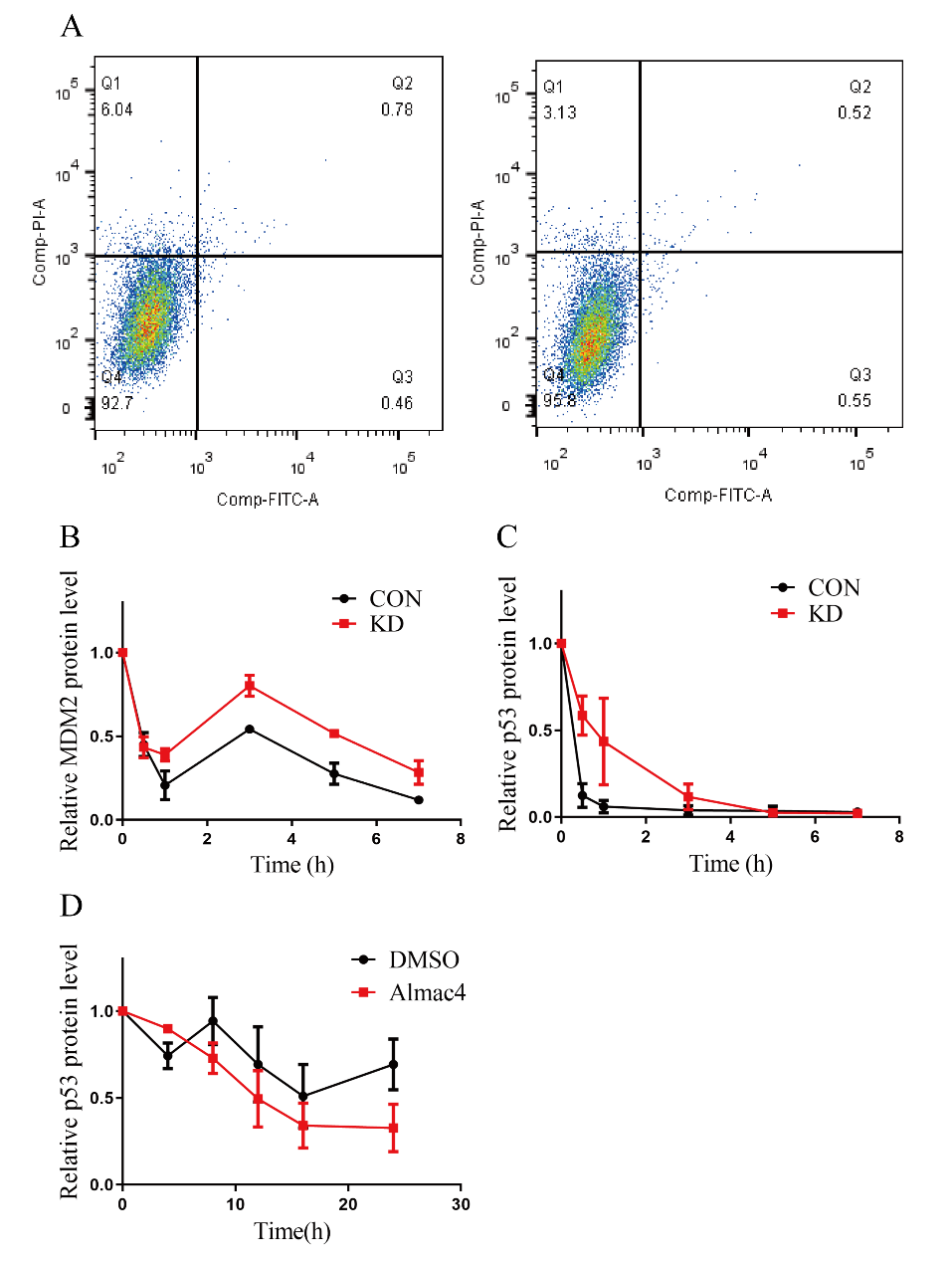


Figure S1 Knockdown of USP7 has no effect on apoptosis. (A) Apoptosis analysis of MKN45 Con and USP7 KD cells; (B) Turnover of MDM2 on USP7 knockdown, the values were normalized on the MDM2 expression in control samples; (C) Turnover of p53 on USP7 knockdown, the values were normalized on the p53 expression in control samples; (D) Turnover of p53 on USP7 inhibition, the values were normalized on the p53 expression in control samples.


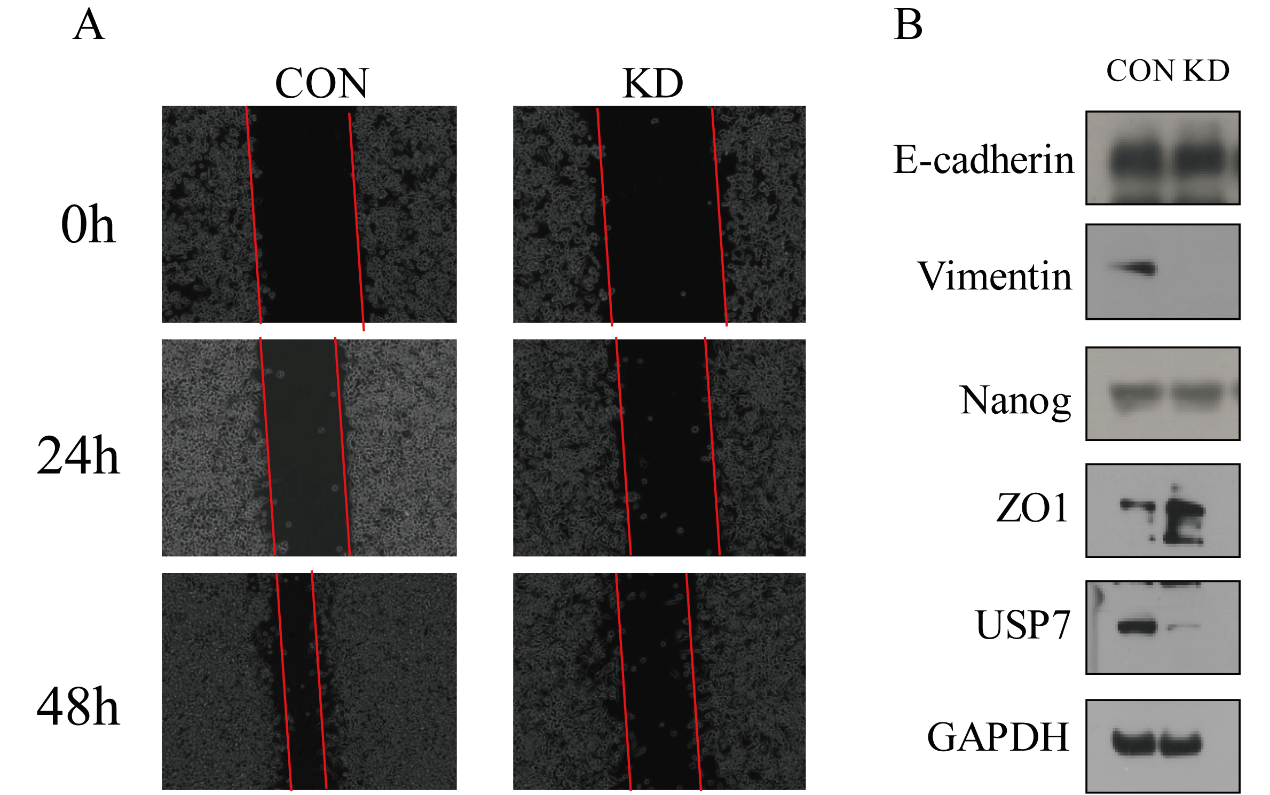


Figure S2 Knockdown of USP7 inhibits migration. (A) Would healing assays were performed to evaluate cell migration after 24h and 48h when USP7 was knockdown in MKN45 cells; (B) Western blot analysis showed the reduced vimentin level when USP7 was knockdown in MKN45 cells.
